# Real-world performance of iFIND-TBR for rapid detection of *Mycobacterium tuberculosis* and rifampicin resistance in China

**DOI:** 10.3389/fmicb.2026.1757837

**Published:** 2026-02-12

**Authors:** Xichao Ou, Huiwen Zheng, Yan Li, Jiaojian Zeng, Lin Huang, Bing Zhao, Feina Li, Hui Xia, Yajie Guo, Ruida Xing, Yuying Chen, Zhonghua Qin, Lixia Zhang, Yanlin Zhao, Yingzi Ma

**Affiliations:** 1 National Key Laboratory of Intelligent Tracking and Forecasting for Infectious Diseases, National Center for Tuberculosis Control and Prevention, Chinese Centre for Disease Control and Prevention, Beijing, China; 2 Laboratory of Respiratory Diseases, Beijing Key Laboratory of Core Technologies for the Prevention and Treatment of Emerging Infectious Diseases in Children, Key Laboratory of Major Diseases in Children, Beijing Pediatric Research Institute, Beijing Children’s Hospital, Ministry of Education, National Clinical Research Center for Respiratory Diseases, National Center for Children’s Health, Capital Medical University, Beijing, China; 3 Institute of Tuberculosis Prevention and Control, Chengde Municipal Center for Disease Control and Prevention (Chengde Municipal Health Supervision Institute), Chengde, China; 4 Center for Accurate Detection of Tuberculosis, Tianjin Haihe Hospital, Tianjin, China

**Keywords:** molecular diagnosis, *Mycobacterium tuberculosis*, rapid, resistance, rifampin

## Abstract

**Objective:**

To evaluate the clinical diagnostic ability of iFIND TBR (iFIND) for *Mycobacterium tuberculosis* (MTB) and resistance to rifampicin (RIF).

**Methods:**

Sputum samples, prospectively collected from patients with suspected pulmonary tuberculosis between November 2023 and December 2024, were used for comprehensive laboratory testing, including smear microscopy, solid culture, Xpert MTB/RIF, iFIND assays, and proportion method drug susceptibility testing (DST).

**Results:**

Among the 452 patients, the positive rates of iFIND (80.31%) and Xpert (76.99%) were significantly higher than those of solid culture (65.49%). Based on solid culture as the reference standard, the sensitivity of iFIND for detection of MTB was slightly higher than that of Xpert, but no statistically significant difference was observed (*p* = 0.157). The sensitivity and specificity of iFIND for detection of MTB were 99.14 and 84.31% relative to the bacteriology reference standard, respectively. Based on the clinical diagnosis results as reference, the higher sensitivity of iFIND than Xpert was observed in detecting MTB (93.46% vs. 90.58%), although the difference was not statistically significant (*p* = 0.162). The rifampicin detection failure rate was significantly higher in low bacterial load specimens (1+) compared to those with moderate/high loads (≥2+) (*p* < 0.001). With the proportion method DST results as reference standard, no statistically significant differences were observed in the sensitivity and specificity between iFIND and Xpert.

**Conclusion:**

The iFIND assay is a rapid and automated assay with sensitivity and specificity comparable to Xpert for early TB diagnosis and drug resistance screening, making it particularly suitable for implementation in primary healthcare settings and general hospitals.

## 1 Introduction

Tuberculosis (TB) remains a global public health emergency, ranking as a leading cause of death from an infectious disease (World Health Organization, 2024). The World Health Organization (WHO) reported an estimated 10.8 million new cases (134 per 100,000 population) in 2023, and China ranked the third among high-TB-burden countries with approximately 741,000 annual cases, including 29,000 multidrug-resistant/rifampicin-resistant TB (MDR/RR-TB) cases, accounting for 7.3% of the global burden (World Health Organization, 2024). Therefore, timely and accurate diagnosis of TB and drug resistance is crucial to eradicate tuberculosis in China.

Despite being the gold standard for TB diagnosis, conventional mycobacterial culture is limited by the requirement of biosafety infrastructure and lengthy culture time (3–8 weeks) (Schumacher et al., 2019). As with low sensitivity and inability to distinguishing non-tuberculous mycobacteria (NTM) from *Mycobacterium tuberculosis* (MTB), the diagnostic utility for smear microscopy is also limited (Steingart et al., 2006). Several nucleic acid amplification tests (NAATs) endorsed by WHO have demonstrated excellent diagnostic performance for MTB over the past decade, particularly the Xpert MTB/RIF (Xpert), which simultaneously detected MTB and rifampicin resistance within 2 h by targeting the *rpoB* gene (World Health Organization, 2021; World Health Organization, 2013; World Health Organization, 2020; Chakravorty et al., 2017; Nathavitharana et al., 2017; Penn-Nicholson et al., 2021). However, its widespread implementation in resource-limited settings remains constrained by infrastructure requirements and economic considerations.

The iFIND TBR (iFIND) assay, based on microfluidic technology, is an all-in-one assay for simultaneously identification TB and rifampicin resistance by targeting IS*6110*, IS*1081* and *rpoB* gene, which integrates nucleic acid extraction, amplification, and detection into a single-use cartridge (Ou et al., 2024). It simplified the cumbersome manual operations with entire results delivered only 85 min. Preliminary studies indicated that the iFIND TBR system is user-friendly, highly accurate, and well-suited for resource-limited settings (Ou et al., 2024). However, clinically validated performance data in real-world settings is unavailable for iFIND TBR. In this study, we employed a head-to-head comparative design to evaluate the performance of iFIND-TBR technology at municipal-level TB prevention and control institutions, which will provide essential evidence to support the scaled implementation of this novel diagnostic platform.

## 2 Methods

### 2.1 Participants

Sputum samples were prospectively collected from presumptive pulmonary tuberculosis (PTB) patients and suspected drug-resistant tuberculosis cases from 10 designated TB healthcare facilities in Chengde City, Hebei Province, between November 2023 and December 2024. The study was approved by the Institutional Review Board of Chengde CDC.

Inclusion criteria were: (1) presumptive PTB cases: newly diagnosed patients with TB-suggestive symptoms (persistent cough with or without sputum production for more than 2 weeks, or chest pain, hemoptysis, night sweats, etc), and have received <2 weeks of anti-tuberculosis treatment within the last 1 month; (2) suspected drug-resistant TB cases meeting any of the following criteria: chronic sputum smear-positive cases, retreatment failure cases, close contacts of rifampicin-resistant TB patients with bacteriologically confirmed TB, new treatment failure cases, relapse or treatment-default cases. Exclusion criteria were: (1) Inadequate/unsuitable specimens: Sputum samples with insufficient volume (<3 mL), gross salivary content, or improper preservation/transport leading to visible contamination or degradation. (2) Incomplete laboratory data: Patients lacking essential laboratory records required for diagnostic classification. (3) Prior anti-TB treatment: Patients who had received >2 weeks of anti-tuberculosis therapy within the last month. (4) Refusal or withdrawal: Patients or their legal guardians who declined to participate or withdrew consent during the study process.

### 2.2 Laboratory methods

All clinical specimens from enrolled patients were transported to Chengde Municipal Center for Disease Control and Prevention for comprehensive laboratory testing, including smear microscopy, solid culture, Xpert MTB/RIF (GeneXpert Dx System 5.1), and iFIND (iFIND studio V1.0.1) assays. Smear microscopy was performed to confirm acid-fast bacilli rapidly (Steingart et al., 2006). Then the specimens were processed using the N-acetyl-l-cysteine-sodium hydroxide method, followed by incubation onto Löwenstein-Jensen (L-J) medium to improve diagnostic accuracy (Peres et al., 2009; Kassaza et al., 2014). Positive cultures were subjected to para-nitrobenzoic acid/thiophene-2-carboxylic acid hydrazide (PNB/TCH) medium to distinguish MTB from NTM. For Xpert MTB/RIF assay, 1 mL processed specimen was thoroughly mixed with 2 mL the provided sample reagent, incubated at room temperature for 10 min, and then loaded into Xpert MTB/RIF cartridges for automated analysis (Boehme et al., 2010). The laboratory technicians conducting the iFIND assay and interpreting the Xpert MTB/RIF results were blinded to the outcomes of the other molecular test, as well as to culture and phenotypic drug susceptibility testing results, until all laboratory data were finalized.

### 2.3 iFIND TBR testing

The assay was performed strictly according to the manufacturer’s instructions (Kunpeng Gene, Beijing). Briefly, 1 mL of sputum sample was added to a pretreatment tube containing 2 × volume of sputum processing solution, followed by vortexed vigorously for 15–30 s, then incubated for 15-min at room temperature to complete liquefaction. Subsequently, 2 mL of processed sample was slowly loaded into the reaction chamber, which was placed into the detection module for automated analysis. The positive detection result of MTB by iFIND can be classified into extremely low (1+) with cycle threshold (Ct) ≥ 29, low (2+) with 25 ≤ Ct < 29, medium (3+) with 19 ≤ Ct < 25, and high (4+) with Ct>19. The iFIND rifampicin resistance results were reported as “resistant,” “sensitive” and “indeterminate.”

### 2.4 Proportion method drug susceptibility testing

A 1 mg/mL bacterial suspension prepared with the standard McFarland tube was diluted to 10–2 mg/mL. Then 0.1 mL aliquot was inoculated onto both rifampicin (40 μg/mL) containing Löwenstein-Jensen (L-J) media and drug-free control media. All cultures were incubated at 37 °C for 4 weeks. Strains were classified as rifampicin-resistant if the proportion of colonies growing on drug-containing medium exceeded 1% of the control medium’s growth. Isolates demonstrating ≤1% growth were considered susceptible (Cambau et al., 2000).

### 2.5 Diagnostic criteria

Comprehensive diagnoses standard (CRS) was based on a combination of laboratory tests, chest radiography, and clinical examinations. The bacteriologically confirmed TB was defined as cases with the positive results if any of smear, culture, or Xpert results were positive. The clinically diagnosed TB was defined as at least 1 symptom and sign, X-ray abnormalities suggestive of tuberculosis, and at least 1 of the following: exposure history of active TB, clinical and radiologic improvement after anti-TB treatment, positive results of tuberculin skin test, or interferon-*γ* release assay (IGRA). Non-TB cases were defined as a definitive diagnosis of another disease.

### 2.6 Discordant results analysis

In cases of discrepancy between iFIND and culture/Xpert method, the bacterial loads in specimens were reviewed. For discordant rifampicin resistance results, initial discordance between iFIND and phenotypic DST was first checked against the Xpert rifampicin resistance result for consistency. Subsequently, all available isolates with discordant resistance results underwent Sanger sequencing of the *rpoB* gene to serve as the definitive arbiter. The final interpretation of rifampicin resistance status was based on the sequencing result.

### 2.7 Statistical analysis

Categorical variables were presented as percentages (%). Between-group differences of paired binomial variables were compared using McNemar’s test, while Pearson’s chi-square test were assessed to compare the performance of iFIND in RIF between low concentration (1+) group and middle or higher concentration (≥2+) group. The diagnostic performance of different molecular techniques was evaluated by calculating sensitivity, specificity, positive predictive value (PPV), and negative predictive value (NPV) using 2 × 2 contingency tables. The 95% confidence intervals for proportions were calculated using the Wilson score method. For the diagnostic odds ratio (DOR), which does not lend itself to the Wilson method, the Wald method was applied to compute its 95% confidence interval. A continuity correction (adding 0.5 to each cell of the contingency table) was used in DOR calculations when zero cells were present to avoid undefined values.

Inter-test agreement was assessed using Cohen’s kappa (*κ*) statistic, interpreted as follows: *κ* = 0.41–0.60, moderate agreement; *κ* = 0.61–0.80, substantial agreement; κ > 0.80, almost perfect agreement. All statistical analyses other than the calculation of DOR with its 95% CI, were performed using SPSS (version 26.0; IBM Corp.). A single-sided *p*-value < 0.05 was considered statistically significant. The Calculation of the DOR was performed with R Studio (version 4.5.2). A higher DOR indicates a higher diagnostic efficiency.

## 3 Results

### 3.1 Baseline characteristics

**Figure 1:**
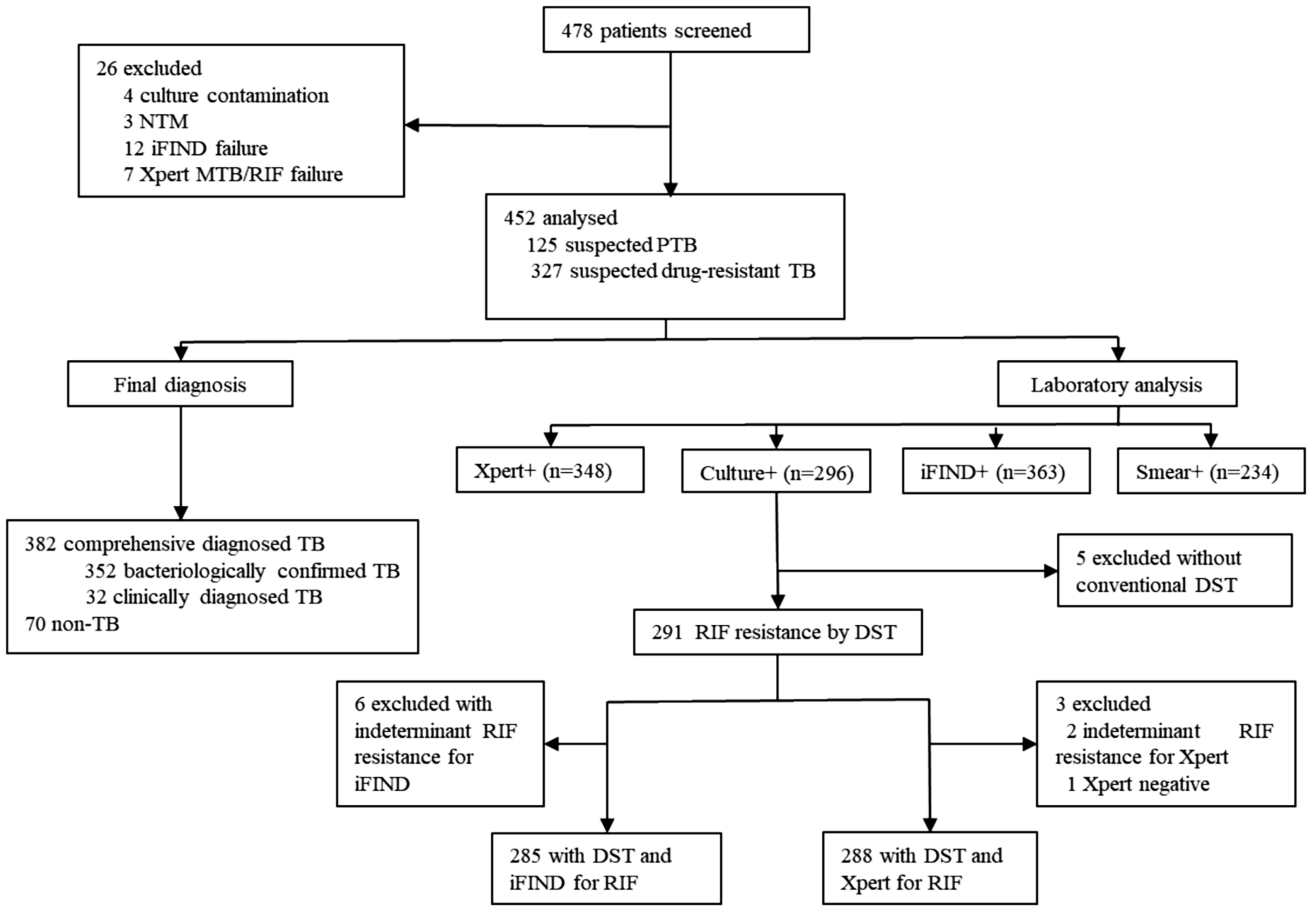
Flow chart of the study population.

A total of 478 patients were initially enrolled in this study. After excluding 3 NTM infection cases, 4 culture contamination cases, 7 Xpert MTB detection failures, and 12 iFIND MTB detection failures, 452 patients including 382 comprehensive diagnosed TB (352 bacteriologically confirmed TB and 30 clinically diagnosed TB), and 70 non-TB cases were eligible for final analysis (Figure 1).

Among the 452 analyzed patients, the MTB positivity rates of iFIND (80.31%, 363/452) and Xpert (76.99%, 348/452) were significantly higher than that of solid culture (65.49%, 296/452) (*χ*^2^ = 25.13, *p* < 0.001 for iFIND; *χ*^2^ = 14.60, *p* < 0.001 for Xpert), respectively.

### 3.2 Performance of iFIND against Xpert for MTB based on solid culture reference

Based on solid culture as the reference standard, the sensitivity of iFIND for the detection of MTB was slightly higher than that of Xpert, but no statistically significant difference was observed (100% vs. 99.32%; *χ*^2^ = 2.01, *p* = 0.157). However, the specificity showed significant difference between iFIND and Xpert (57.05% vs. 65.38%; *χ*^2^ = 8.89, *p* = 0.003). The 67 cases showed discordant results between iFIND and culture methods, all demonstrating iFIND-positive but culture-negative outcomes, of which 76.12% (51/67) were Xpert MTB-positive and 91.04% (61/67) were clinically diagnosed as TB. Both iFIND and Xpert diagnostic methods showed substantial agreement with solid culture results, with Kappa values >0.61 (Table 1).

**Table 1:** Performance of iFIND against Xpert for MTB based on solid culture reference.

Method	Culture		Total	Sensitivity (%, 95CI)	Specificity (%, 95CI)	PPV (%, 95CI)	NPV (%, 95CI)	*Kappa*
	Pos	Neg						
iFIND								
Pos	296	67	363	100.00 (98.72–100.00)	57.05 (49.21–64.56)	81.54 (77.23–85.20)	100.00 (95.86–100.00)	0.64
Neg	0	89	89					
Total	296	156	452					
Xpert								
Pos	294	54	348	99.32 (97.57–99.81)	65.38 (57.63–72.40)	84.48 (80.30–87.91)	98.08 (93.26–99.47)	0.70
Neg	2	102	104					
Total	296	156	452					
Smear								
Pos	204	30	234	82.59 (77.37–86.81)	80.77 (73.88–86.18)	87.18 (82.29–90.87)	74.56 (67.49–80.53)	0.62
Neg	43	126	169					
Total	247	156	403					

Smear microscopy was not performed on 49 specimens, as these were extrapulmonary samples for which smear microscopy is not routinely recommended dueto its well-documented low sensitivity in such sample types.

### 3.3 Accuracy of iFIND against bacteriological evidence and Xpert for MTB

The sensitivity and specificity of iFIND for the detection of MTB were 99.14% (95% CI: 97.6–99.8%) and 84.31% (95% CI: 76.0–90.6%) relative to the bacteriological reference standard, respectively. The agreement was almost perfect concordance between iFIND and the bacteriology standard (*κ* = 0.87, 95% CI: 0.82–0.92), supported by the DOR of 566.86 (95% CI: 186.80–2559.98) (Table 2).

**Table 2:** Accuracy of iFIND against bacteriological evidence and Xpert for MTB.

Method	Xpert		Total	Sensitivity (%, 95CI)	Specificity (%, 95CI)	PPV (%, 95CI)	NPV (%, 95CI)	*Kappa*	*DOR*
	Pos	Neg							
iFIND									
Pos	345	18	363	99.14 (97.50–99.71)	82.69 (74.29–88.76)	95.04 (92.30–96.84)	96.63 (90.55–98.85)	0.86	502.86 (167.73, 2214.02)
Neg	3	86	89						
Total	348	104	452						

	Bacteriological evidence								
	Pos	Neg							
iFIND									
Pos	347	16	363	99.14 (97.51–99.71)	84.31 (76.03–90.11)	95.59 (92.96–97.27)	96.63 (90.55–98.85)	0.87	566.56 (186.80, 2559.98)
Neg	3	86	89						
Total	350	102	452						

Using Xpert MTB/RIF as reference standard, the sensitivity of iFIND assay for MTB was 99.14% (95% CI: 97.6–99.8%) and specificity was 82.69% (95% CI: 74.1–89.3%). The agreement between the two assays was almost perfect concordance, with a Kappa value of 0.86 (95% CI: 0.81–0.91), supported by the DOR of 502.86 (95% CI: 167.73–2214.02) (Table 2). For the 3 culture-positive/iFIND-negative specimens, 66.7% (2/3) showed “very low” bacterial load and 33.3% (1/3) showed “low” load by Xpert quantification. Conversely, among 18 iFIND-positive/Xpert-negative specimens, 77.8% (14/18) were classified as “very low positive” (1+) by iFIND.

### 3.4 Analysis of iFIND and Xpert compared with clinical diagnosis for MTB

Based on the clinical diagnosis results as reference, the higher sensitivity of iFIND than Xpert was observed in detecting MTB (93.46% vs. 90.58%), the difference was statistically significant (*χ*^2^ = 8.07, *p* = 0.005). The specificity of iFIND was lower than that of Xpert (91.43% vs. 97.14%), but there was also no significant difference between iFIND and Xpert (*χ*^2^ = 2.12, *p* = 0.145). A high degree of consistency was observed with between clinical diagnosis and both iFIND (*κ* = 0.76) and Xpert (*κ* = 0.73), which was further supported by their diagnostic odds ratios of 152.32 (95% CI: 60.11–386.01) and 326.78 (95% CI: 76.85–1389.55), respectively (Table 3).

**Table 3:** Analysis of iFIND, Xpert and culture compared with clinical diagnosis for MTB.

Method	Clinical diagnosis		Total	Sensitivity (%, 95CI)	Specificity (%, 95CI)	PPV (%, 95CI)	NPV (%, 95CI)	*Kappa*	*DOR*
	TB	Non-TB							
iFIND									
Pos	357	6	363	93.46 (90.52–95.53)	91.43 (82.53–96.01)	98.35 (96.44–99.24)	71.91 (61.82–80.19)	0.76	152.32 (60.11–386.01)
Neg	25	64	89						
Total	382	70	452						
Xpert									
Pos	346	2	348	90.58 (87.23–93.12)	97.14 (90.17–99.21)	99.43 (97.93–99.84)	65.38 (55.84–73.83)	0.73	326.78 (76.85–1389.55)
Neg	36	68	104						
Total	382	70	452						
Culture									
Pos	296	0	296	77.49 (73.04–81.39)	100.00 (94.80–100.00)	100.00 (98.72–100.00)	44.87 (37.28–52.71)	0.52	483.31 (29.63–7883.87)
Neg	86	70	156						
Total	382	70	452						

### 3.5 Comparison of iFIND and Xpert in detecting rifampicin resistance

Among 363 iFIND MTB-positive patients, 21 (5.8%) cases yielded “indeterminate” RIF-resistance results, of which 95.2% (20/21) had extremely low bacterial load (1+) and 4.8% (1/21) had low bacterial load (2+). The RIF detection failure rate was significantly higher in low bacterial load specimens (1+) compared to those with moderate/high loads (≥2+) (*χ*^2^ = 50.44, *p* < 0.001) (Supplementary Table 1).

With the proportion method drug susceptibility test results as the reference standard, the sensitivity and specificity of iFIND in detecting rifampicin resistance were 98.10% (103/105) and 97.22% (175/180), respectively. The sensitivity and specificity of Xpert in detecting rifampicin resistance were 98.11% (104/106) and 95.60% (174/182), respectively. There were no statistically significant differences in the sensitivity and specificity between iFIND and Xpert (*χ*^2^ = 0.00, *p* = 0.992; *χ*^2^ = 68, *p* = 0.408) (Table 4). Of 7 cases with discordant rifampicin resistance results between iFIND and DST, while showing complete concordance between iFIND and Xpert resistance profiles, 2 iFIND-sensitive/phenotypically-resistant cases were confirmed as sensitive by sequencing. Of 5 iFIND-resistant/phenotypically-sensitive cases, 3 successfully sequenced isolates were all harboring *rpoB* resistance-conferring mutations, while 2 isolates failed resuscitation (Supplementary Table 2).

**Table 4:** Comparison of iFIND and Xpert in detecting rifampicin resistance with proportion method DST.

Method	Proportion method		Total	Sensitivity (%, 95CI)	Specificity (%, 95CI)	PPV (%, 95CI)	NPV (%, 95CI)	*Kappa*	*DOR*
	Resistance	Susceptibility							
iFIND									
Resistance	103	5	108	98.10 (93.32–99.48)	97.22 (93.66–98.81)	95.37 (89.62–98.01)	98.87 (95.97–99.69)	0.95	1802.5 (343.48–9458.98)
Susceptibility	2	175	177						
Total	105	180	285						
Xpert									
Resistance	104	8	112	98.11 (93.38–99.48)	95.60 (91.57–97.76)	92.86 (86.54–96.34)	98.86 (95.95–99.69)	0.93	1,131 (235.67–5427.71)
Susceptibility	2	174	176						
Total	106	182	288						

Taking the Xpert test results as the reference standard, the iFIND test demonstrated a sensitivity of 98.43% and a specificity of 100% for rifampicin resistance detection. This corresponded to a near-perfect agreement (*κ* = 0.99) and a DOR of 20431.4 (95% CI: 972.94–429050.3), indicating excellent concordance between the two methods. (Table 5).

**Table 5:** Accuracy of iFIND against Xpert in detecting rifampicin resistance.

Method	Xpert		Total	Sensitivity (%, 95CI)	Specificity (%, 95CI)	PPV (%, 95CI)	NPV (%, 95CI)	*Kappa*	*DOR*
	Resistance	Susceptibility							
iFIND									
Resistance	125	0	125	98.43 (94.44–99.57)	100.00 (98.14–100.00)	100.00 (97.02–100.00)	99.02 (96.51–99.73)	0.99	20431.4 (972.94–429050.3)
Susceptibility	2	203	205						
Total	127	203	330						

## 4 Discussion

To address critical gaps in global tuberculosis control, there is an urgent need for novel diagnostic solutions that can overcome the limitations of current methods by delivering enhanced diagnostic accuracy, operational advantages, and implementation feasibility (Seki et al., 2018; MacLean et al., 2020). iFIND, an automatic molecular point-of care testing (POCT) system, provides an option in accurately and rapidly for TB diagnosis and resistance detection. The specific targets are crucial for diagnosis, and IS*6110* was considered as the most commonly target for detecting MTB (Eisenach et al., 1990; Thierry et al., 1990). However, it was reported to be missing in some clinical samples, resulting in false-negative (Nghiem et al., 2015) and false-positive results (Sankar et al., 2011). Therefore, iFIND combined IS*6110* with another insertion sequence IS1081 (Vansoolingen et al., 1992; Collins and Stephens, 1991) based on nested real-time fluorescent quantitative PCR technology to eliminate the false-negative results of MTB diagnosis. Moreover, iFIND utilized five distinct molecular beacons specifically designed to the rifampicin resistance-determining region (RRDR) of the *rpoB* gene, using the melting curve method to detect rifampin resistance. Preliminary research results on frozen clinical samples showed that the iFIND detection system is simple to operate and has high accuracy (Ou et al., 2024).

This in real world head-to-head evaluation demonstrated that iFIND achieved a sensitivity of >98% for MTB detection compared with the solid culture reference and bacteriologically TB standard, respectively. The observed discordance, specifically bacteriology-positive but molecular-negative results, reflects a well-recognized diagnostic challenge in global TB control. A large operational study in Myanmar reported that 4% of smear-positive patients were Xpert-negative, with low bacterial load (scanty/1+) and advanced age (≥65 years) identified as key contributing factors (Phyu et al., 2018), aligning closely with our findings. In our cohort, all culture-positive but iFIND-negative specimens exhibited low or very low bacterial loads upon Xpert quantification. Conversely, 91.04% cases with iFIND-positive but culture-negative outcomes were clinically diagnosed as TB, indicating the comparability sensitivity in MTB detection for iFIND. According to the WHO’s Target Product Profile (TPP) (World Health Organization, 2024), the optimal sensitivity requires≥95% for MTBC detection on sputum-based assays. And the sensitivity of iFIND for the detection of MTB was significantly higher than that of Xpert compared with clinically diagnosis standard. Therefore, iFIND was a robust tool for paucibacillary samples, such as those from HIV/TB co-infected, extrapulmonary TB or pediatric TB patients. Though there was no statistically significant difference, the sensitivity of iFIND for the detection of MTB was slightly higher than that of Xpert compared with solid culture reference. And the discordant results between iFIND and Xpert MTB assays predominantly occurred in specimens with low bacterial loads, which are more susceptible to minor perturbations, such as sample degradation during transport, inadequate homogenization, and processing delays, resulting in false-negative results.

It is important to note that this study employed multiple reference standards to ensure a comprehensive and clinically relevant evaluation of iFIND’s diagnostic performance. In clinical practice, especially in high-TB-burden settings, no single test is perfect or universally available. The traditional “gold standard” of solid culture, while highly specific, may yield false negatives in paucibacillary cases or be inaccessible in resource-limited settings. By adopting a bacteriological standard, we aimed to maximize sensitivity for case detection, reflecting the real-world clinical strategy of utilizing all available laboratory evidence. This approach provides a performance estimate most relevant to clinicians, who must act upon any positive diagnostic result. Conversely, the use of clinical diagnosis as a comparator enabled us to evaluate test performance in the challenging subgroup of culture-negative patients who still require treatment, allowing us to assess the utility of iFIND across the full spectrum of presumptive TB. Our analytical focus was directed toward comparing newer, more sensitive molecular assays against the most relevant and rigorous benchmarks available. Consequently, directly comparing with smear positivity holds limited utility, given that smear microscopy exhibits low sensitivity and is frequently negative in paucibacillary and extrapulmonary TB, which constitute a substantial proportion of clinically diagnosed cases. A separate comparison would primarily underscore the well-established limitations of smear microscopy in these populations, offering little novel insight into the performance of the iFIND assay.

In this study, the diagnostic efficiency of iFIND was evaluated using the DOR. As a comprehensive metric that integrates both sensitivity and specificity, the DOR reflects the ratio of the odds of a positive result in diseased individuals to the odds in non-diseased individuals, thereby providing a single statistical measure of overall test performance. A higher DOR generally indicates better diagnostic accuracy. Regardless of the reference standard applied, iFIND consistently yielded high DOR values, demonstrating its robust diagnostic efficiency. Moreover, shorter diagnostic turnaround time (<2 h) for iFIND enable clinical decision-making at municipal TB control centers on the same-day of patient visit, making it particularly advantageous in resource-limited settings. And the automated computer-based interpretation eliminates subjective errors associated with manual result analysis. Although the iFIND assay exhibited a slightly higher failure rate (2.51%, 12/478) compared to Xpert (1.46%, 7/478), this difference was not statistically significant (*p* > 0.05). The primary reasons for test failure may contribute to technical errors during initial processing steps, which are unlikely to pose a major barrier to the deployment of iFIND in primary healthcare or general hospital laboratories.

The iFIND TBR assay also facilitates detection of RIF resistance mutations within RRDR. In this study, iFIND demonstrated sensitivities of 98.10% for RIF resistance, meeting the WHO TPP targets of ≥95% for RIF (World Health Organization, 2024), which is effective in diagnosing clinical rifampicin resistance. However, the sensitivity of iFIND for rifampicin resistance detection is lower than that of MTB diagnosis, indicating that MTB-positive with indeterminate rifampicin resistance cases may occurred. Moreover, our data revealed a strong bacterial load-dependent performance pattern (Supplementary Table 1), where specimens with low-level MTB positivity (1+) exhibited lower resistance detection rates compared to those with a higher bacterial load (≥2+). This observation aligns with established literature that samples with low bacterial load are likely to result in inconsistent molecular RIF susceptibility results (Deng et al., 2023; Huo et al., 2020). However, there are some samples with discordant results between iFIND TBR and phenotypic DST, but consistent with the Xpert assay. The 2 iFIND-sensitive/phenotypically-resistant cases were confirmed as sensitive by sequencing, which may be due to mutations outside the RRDR region, potentially leading to undetected mutations for iFIND. And 3 iFIND-resistant/phenotypically-sensitive cases (Supplementary Table 2) included one hetero-resistance and two cases with mutations in *rpoB*_L511P and L533P, respectively, suggesting that these two mutations may be associated with low-level resistance to RIF, which falls within or below the critical value for the proportional method of RIF. Therefore, iFIND offers clinically guidance for tuberculosis management, particularly in treatment-experienced patients where resistance patterns may be complex. Moreover, two iFIND-sensitive/Xpert-resistant cases were confirmed as heteroresistant mutations by sequencing analysis. This discrepancy may be attributed to the methodological and interpretation criteria differences, with Xpert using PCR fluorescence probe method, while iFIND uses melting curve method. In addition, uneven distribution of mutant and wild-type bacilli in clinical specimens might lead to differences in the results of the two tests.

This study has several limitations. First, this study is limited to the Chengde, Hebei Province, which may not fully represent the genetic diversity of *Mycobacterium tuberculosis* across China. To ensure broader applicability, future studies should be validated through multicenter studies and geographically diverse cohorts. Second, while this study provides valuable data on iFIND performance for pulmonary tuberculosis diagnosis, its applicability to extrapulmonary TB and pediatric cases remains to be established. Third, the exclusion of tests that failed due to technical or sample-related reasons, though low in number, may lead to an underestimation of real-world operational challenges. Fourth, we did not include a formal cost-effectiveness analysis or an assessment of long-term health system impact, which are crucial for guiding policy and implementation. Finally, inter-operator variability in test performance was not systematically evaluated.

## 5 Conclusion

This study demonstrated that the iFIND assay is a rapid and automated assay with high sensitivity and specificity comparable to Xpert for early TB diagnosis and drug resistance screening, making it particularly suitable for implementation in primary healthcare settings and general hospitals.

## Data Availability

The original contributions presented in the study are included in the article/Supplementary material, further inquiries can be directed to the corresponding authors.
