# Supplementary material for: Real-world performance of iFIND-TBR for rapid detection of Mycobacterium tuberculosis and rifampicin resistance in China
Source: Front Microbiol. 2026 Feb 12;17:1757837. doi: 10.3389/fmicb.2026.1757837 (PMC12937132; doi:10.3389/fmicb.2026.1757837)
Supplement: Supplementary file 1 [file Table_1.DOCX]

Supplementary table 1. Correlation between bacterial load the RIF-resistance results

| iFIND MTB | TBR RIF | | | |
| --- | --- | --- | --- | --- |
|  | Resistance | Susceptibility | Indeterminate | Total |
| 1+ | 24 | 61 | 20 | 105 |
| 2+ | 34 | 63 | 1 | 98 |
| 3+ | 42 | 62 | 0 | 104 |
| 4+ | 29 | 33 | 0 | 62 |
| Total | 129 | 219 | 21 | 369 |

Supplementary table 2. iFIND-resistant/phenotypically-sensitive cases

| Number | Proportion method | iFIND | Mutation |
| --- | --- | --- | --- |
| R-2078 | Susceptibility | Resistance | L511P |
| R-2103 | Susceptibility | Resistance | L511P、H526L、S531L |
| R-3039 | Susceptibility | Resistance | L533P |
